# The Hybrid Multiple-Criteria Decision-Making Model for Home Healthcare Nurses’ Job Satisfaction Evaluation and Improvement

**DOI:** 10.3389/ijph.2022.1604940

**Published:** 2022-09-28

**Authors:** YanJiao Wang, YaQin Ye, Yanjun Jin, Yen-Ching Chuang, Ching-Wen Chien, Tao-Hsin Tung

**Affiliations:** ^1^ Xiamen Cardiovascular Hospital, Xiamen University, Xiamen, China; ^2^ Institute for Hospital Management, Tsing Hua University, Shenzhen Campus, Shenzhen, China; ^3^ Sanmen People’s Hospital of Zhejiang, Sanmen, China; ^4^ Department of Nursing, Taizhou Hospital of Zhejiang Province affiliated to Wenzhou Medical University, Taizhou, China; ^5^ Institute of Public Health and Emergency Management, Taizhou University, Taizhou, China; ^6^ Business College, Taizhou University, Taizhou, China; ^7^ Evidence-Based Medicine Center, Taizhou Hospital of Zhejiang Province Affiliated to Wenzhou Medical University, Linhai, China

**Keywords:** home healthcare nurse job satisfaction scale (HHNJS), job satisfaction evaluation and improvement, consistent fuzzy preference relations (CFPRs), importance-performance analysis (IPA), multiple criteria decision-making (MCDM)

## Abstract

**Objectives:** To investigate and evaluate the key factors related to job satisfaction performance of home healthcare nurses (HHNs).

**Methods:** A total of 31 HHNs from three community hospitals in Zhejiang province were invited to participate in the study. They completed a questionnaire survey based on the home healthcare nurse job satisfaction scale (HHNJS) from February to March 2022. Consistent fuzzy preference relation (CFPR) methods and important-performance analysis (IPA) were used to obtain the attribute weights and performance for HHNs job satisfaction.

**Results:** The results showed that the attributes of *C*_13_, *C*_14_, *C*_15_, *C*_23_, *C*_24_, *C*_42_, *C*_51_, and *C*_52_ were key factors influencing HHNs job satisfaction.

**Conclusion:** The hybrid multiple-criteria decision-making (MCDM) model can help home-healthcare-agency administrators better understand the key factors related to HHNs job satisfaction and establish reasonable improvement strategies.

## Introduction

The coronavirus disease (COVID-19) is a highly communicable disease that has led to more than 257 million infections and 5000 thousand deaths worldwide [1]. The rapid spread of COVID-19 has considerably strained the global healthcare system and increased the workload and pressure on healthcare workers [2, 3]. During this pandemic, nurses are the primary participants in COVID-19 patient management. Their role encompasses patient assessment and classification, patient care, and specimen collection [4]. Home healthcare focuses primarily on the disabled and older adults vulnerable to COVID-19. Therefore, home healthcare nurses (HHNs) play an essential role in preventing the spread of COVID-19 [5, 6].

In addition to their standard care for patients without COVID-19 during the pandemic, nurses have worked hard to care for sick patients, provide comfort in the face of death, and educate the public about protection measures to stop the SARS-CoV-2 spread [7]. Many nurses have been burdened by increased workload and stress while facing a high risk of COVID-19 infection and death [8]. More than 2200 nurses have died from COVID-19, and this figure will undoubtedly grow [9]. Before the pandemic, nursing shortages and high turnover rates were already a problem in many countries. This shortage has been exacerbated by the COVID-19 pandemic [10, 11]. Job satisfaction is an important factor influencing nurse retention and quality of care [12]. The turnover rate of HHNs is higher than that of total registered nurses [13], while decreased job satisfaction is related to turnover and low job desirability for home healthcare nursing [14]. With an increase in the aging population, the demand for home healthcare has been steadily increasing [15]. Hence, knowing the factors that impact HHNs job satisfaction and accurately measuring job satisfaction can help home-healthcare-agency administrators retain the nursing workforce [16].

The Home Healthcare Nurse Job Satisfaction Scale (HHNJS) is a reliable and valid instrument for measuring the job satisfaction of HHNs. It includes eight subscales—four focus on relationships and four on other work aspects [17]. Some studies have used the HHNJS to assess job satisfaction among HHNs; for example, Ellenbecker et al. used the HHNJS to measure the job satisfaction of 340 HHNs from 10 agencies. The findings showed that relationships with patients, autonomy, and professional pride contributed most to HHNs job satisfaction [18]. Li et al. used the HHNJS to assess the job satisfaction of 40 HHNs, and the responses to most items were “strongly agree” or “agree” [19]. However, the attribute weights of HHNJS as they relate to improvement in HHNs job satisfaction have not been thoroughly investigated.

Our study established a multiple-criteria decision-making (MCDM) model and applied it to the HHNs job satisfaction analysis. Consistent fuzzy preference relation (CFPR) was initially used to obtain HHNJS attribute weights before the importance-performance analysis (IPA) method was applied to analyze the job satisfaction of HHNs performance.

This study is structured as follows: *Methods* section introduces the HHNJS survey questionnaire and illustrates the CFPRs calculation procedures and IPA methods, *Results* section outlines the results of applying the hybrid MCDM model to the HHNs job satisfaction assessment, *Discussion* section discusses the results and limitations of the study, and *Conclusion* section presents our conclusions.

## Methods

### Study Design

**FIGURE 1:**
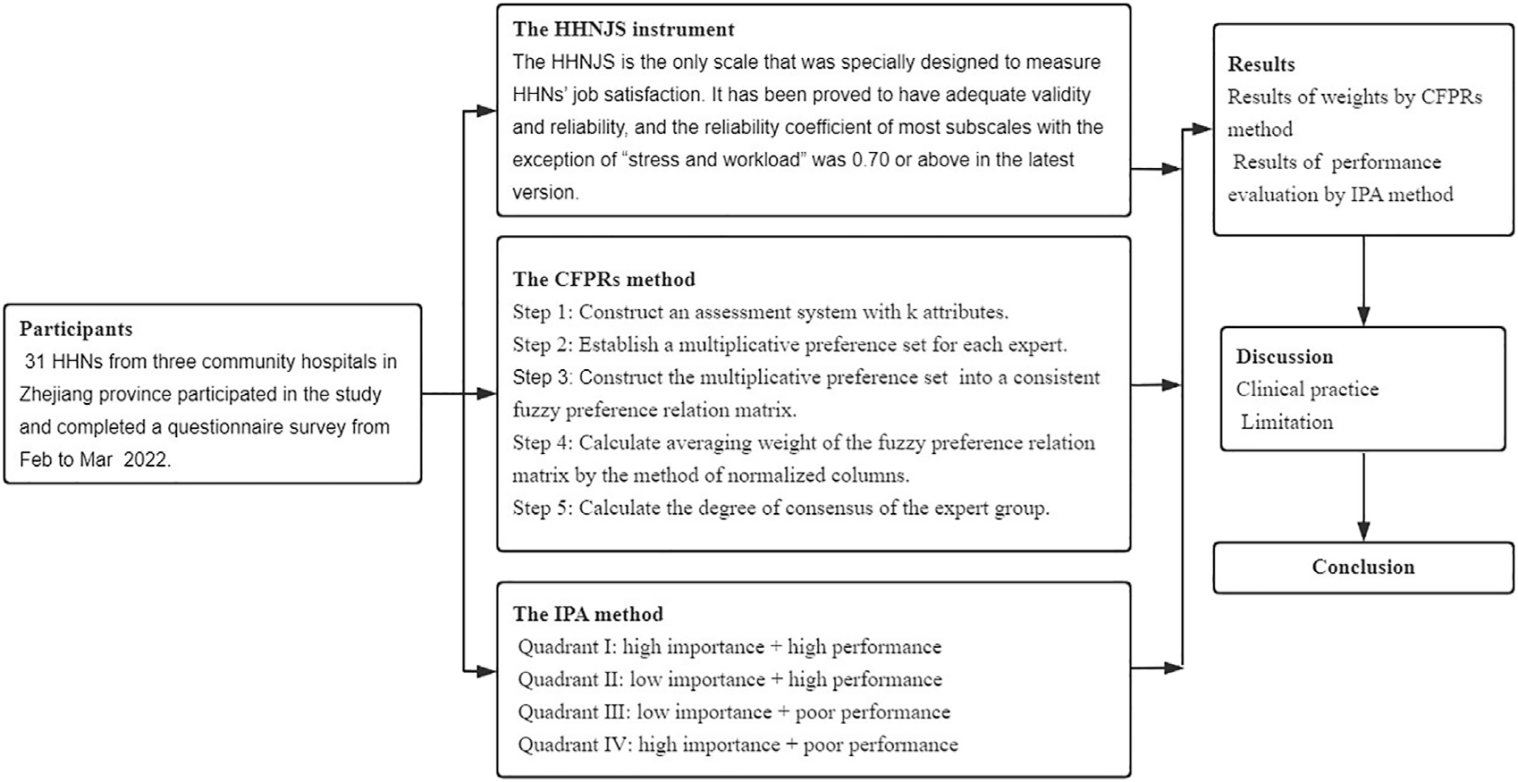
The study design flow chart (China, 2022).

This study aims to develop an MCDM model and apply it to the community hospitals in the present study. The HHNJS instrument is an evaluation tool that includes eight subscales. The dimensions and criteria weights were then constructed using the CFPRs method. This model was ultimately used to evaluate and improve the satisfaction of HHNs in the community hospitals in the present study. The study flow chart is shown in Figure 1.

### The Home Healthcare Nurse Job Satisfaction Scale Instrument

The HHNJS is the only scale specifically designed to measure HHNs job satisfaction [20]. It was developed by Ellenbecker and her colleagues and has been revised several times to enhance its psychometric properties [17, 20–22]. Previous empirical studies regarding job satisfaction and Neal’s theory of HHNs practice form the theoretical framework of the instrument [23–27]. The HHNJS has been proved to have adequate validity and reliability. The reliability coefficient of most subscales, except for “stress and workload,” was 0.70 or above in the latest version [17, 22].

The HHNJS comprises 30 items to measure the level of job satisfaction in eight components: “relationship with patients (*C*_1_),” “relationship with peer (*C*_2_),” “professional pride (*C*_3_),” “salary and benefit (*C*_4_),” “relationship with physician (*C*_5_),” “relationship with organization (*C*_6_),” “autonomy and control (*C*_7_)” and “stress and workload (*C*_8_)” [14]. Each of the 30 items is rated on a 5-point Likert-type scale from 1 to 5 (“strongly disagree” to “strongly agree”). The components of the HHNJS instrument are listed in Table 1.

**TABLE 1:** The home healthcare nurse job satisfaction scale (United States, 2001).

Dimension	Criteria
Relationship with patients (*C*_1_)	Patients are satisfied with the care that I provide (*C*_11_)
	The relationships that I have established with patients are rewarding (*C*_12_)
	I have helped patients maintain or improve their quality of life (*C*_13_)
	My work is important and worthwhile (*C*_14_)
	The patient care that I provide adheres to my professional standards (*C*_15_)
Relationship with peer (*C*_2_)	The support I have from my nursing peers is a positive aspect of my job (*C*_21_)
	I can communicate comfortably with the nurses I work with (*C*_22_)
	There is a good amount of collegiality among the nurses I work with (*C*_23_)
	I have peers whom I can rely on and turn to if necessary (*C*_24_)
Professional pride (*C*_3_)	If I had to do it over again, I would choose home healthcare as my area of practice (*C*_31_)
	I would commend my job to another health care professional (*C*_32_)
	I am proud to talk to people about the work I do (*C*_33_)
Salary and benefit (*C*_4_)	My present salary is satisfactory (*C*_41_)
	An upgrading of the pay scales at this agency is needed (*C*_42_)
	Nursing salaries at other agencies are better than salaries at this agency (*C*_43)_
	The benefit package at this agency is satisfactory to me (*C*_44_)
Relationship with physician (*C*_5_)	Physicians value my input on the status of their home healthcare patients (*C*_51_)
	I am treated as a professional colleague by physicians (*C*_52_)
Relationship with organization (*C*_6_)	I am satisfied with the professional relationship that I have with nursing administration at this agency (*C*_61_)
	I have the power to generate change in organizational policy at this agency (*C*_62_)
	I have the opportunity to grow and develop as a professional nurse within this agency (*C*_63_)
Autonomy and control (*C*_7_)	I am able to adjust the hours of my work if needed (*C*_71_)
	I have more flexibility in my hours of work than nurses in other practice settings (*C*_72_)
	I have sufficient control over scheduling my time (*C*_73_)
	I have the independence to make important decisions in my day-to-day work (*C*_74_)
Stress and workload (*C*_8_)	At times I am overwhelmed by all the work I have to do (*C*_81_)
	I could deliver better patient care if I had more time (*C*_82_)
	I am able to meet the demands of my job (*C*_83_)
	I am able to cope with the increased demands for documentation in home care (*C*_84_)
	Sometimes I get frustrated because all my activities are programmed for me (*C*_85_)

### The Consistent Fuzzy Preference Relations Method

AHP is a commonly used MCDM method to evaluate the relative attribute weights by performing pairwise comparisons [28]. The decision-makers compare a pair of attributes at a time. While there are *k* attributes in the evaluation system, it needs to be compared $c_{2}^{K}=k\left(k-1\right)/2$ times. Too many comparisons between attribute pairs may result in inconsistencies, including primacy, recency, and the bandwagon effect [28, 29]. Hence, Herrera-Viedma et al. developed the CFPRs method to cope with the many pairwise comparisons and inconsistencies in AHP [30]. This approach requires only k−1 pairwise comparisons when k attributes are involved in the evaluation system [31]. The remaining (k−1)(k/2−1) comparisons can be computed using the CFPR method, which only refers to simple calculative procedures and ensures consistent comparison results [32]. CFPRs are easy to use and offer a practical way to obtain the attribute weights in the MCDM method [33]. Thus, they have been applied to hazard assessment [34], supplier selection [31], logistic outsourcing [35], and many other topics. The calculation procedure for this method is shown in the Supplementary Appendix [30, 31, 36].

### The Important-Performance Analysis Method

**FIGURE 2:**
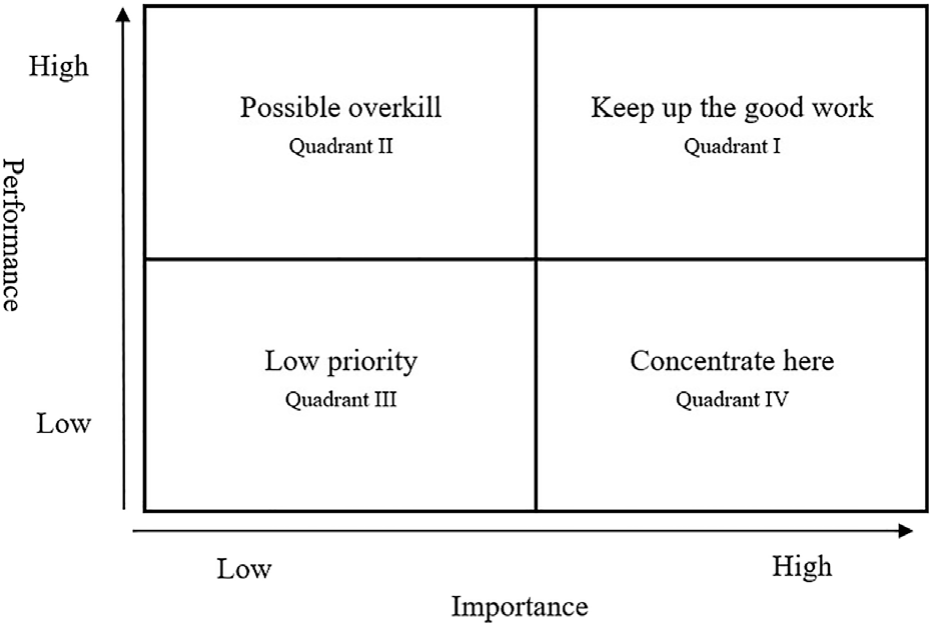
Important-performance analysis four quadrant diagram (United States, 1977).

The IPA method was first proposed by Martilla et al. in 1977 in marketing [37] and has been applied to many academic fields, such as dental practice [38], hospital quality [39], education [40], and tourism [41]. The IPA method is a useful research technique to help decision-makers evaluate customer satisfaction with products or services [42]. IPA provides valuable information by measuring the importance and performance of each attribute and dividing the attributes into four quadrants, as shown in Figure 2.

In quadrant I—keep up the good work—the attributes are both highly important and high performance. This means that these attributes are organizational strengths, and decision-makers should maintain resource input. In other words, nursing department decision-makers should maintain these attributes to maintain acceptable levels of HHNs job satisfaction.

Attributes in the “possible overkill” quadrant have high customer satisfaction with performance but are unimportant. Resources invested in these attributes can be reduced and should be applied elsewhere. In the short term, nursing decision-makers can temporarily reduce their attention to these attributes and use related resources for Quadrant IV attributes to improve job satisfaction.

Attributes in the “low priority” quadrant have low importance, poor performance, and can be temporarily ignored. Compared with other attributes, nursing department decision-makers can directly identify the resources related to these and other attributes that can improve job satisfaction.

Attributes located in “concentrate here” are highly important but have poor performance, which indicates that the attributes require immediate improvement, and decision-makers should focus on them. Nursing decision-makers should increase their satisfaction with these attributes and divert resources from Quadrants II or III to these attributes.

### Data Collection

Thirty-one HHNs from three community hospitals in Zhejiang Province participated in the study and completed a questionnaire survey from February to March 2022. The questionnaire was comprised of three parts: the first evaluated the relative importance of the attributes, the second was a self-evaluation of job satisfaction with home healthcare, and the third covered participant demographics. 100% of participants were female, and 48% had a bachelor’s degree. 58% percent of nurses were younger than 30 years old, and most were senior nurses. 61% of participants had been nursing for less than 10 years, while 81% had worked in home healthcare nursing for no more than 5 years. Table 2 shows the participant information.

**TABLE 2:** The background and characteristics of 31 home healthcare nurses (China, 2022).

Characteristics	Value (%)
Gender	
Male	0 (0%)
Female	31 (100%)
Education	
Technical school education	2 (6%)
Junior college	14 (45%)
Bachelor	15 (48%)
Age	
<30	18 (58%)
30–39	9 (29%)
40 and above	4 (13%)
Professional title	
Senior nurse	22 (71%)
Supervisor nurse	8 (26%)
Co-chief nurse	1 (3%)
Yeas of nursing service	
Under 10 years	19 (61%)
10–15	6 (19%)
15–20	5 (16%)
>20	1 (3%)
Years of home healthcare service	
≤5	25 (81%)
>5	6 (19%)

## Results

### The Attribute Weight Results

Table 3 lists the attribute weights of the 31 HHNs based on the HHNJS scale. The confidence level of the weighted results was 0.7%, lower than 5%.

**TABLE 3:** Weights for dimensions and criteria (China, 2022).

Dimensions	Local weight	Ranking	Criteria	Local weight	Ranking	Global weight	Ranking
C_1_	0.1731	1	C_11_	0.1207	5	0.0209	24
			C_12_	0.1478	4	0.0256	19
			C_13_	0.1808	3	0.0313	15
			C_14_	0.2663	2	0.0461	7
			C_15_	0.2845	1	0.0493	4
C_2_	0.1461	3	C_21_	0.0935	4	0.0137	29
			C_22_	0.2021	3	0.0295	17
			C_23_	0.3253	2	0.0475	5
			C_24_	0.3791	1	0.0554	2
C_3_	0.1284	4	C_31_	0.2051	3	0.0263	18
			C_32_	0.3657	2	0.0469	6
			C_33_	0.4292	1	0.0551	3
C_4_	0.1463	2	C_41_	0.1086	4	0.0159	27
			C_42_	0.2951	3	0.0432	12
			C_43_	0.3002	1	0.0439	9
			C_44_	0.2961	2	0.0433	10
C_5_	0.1095	5	C_51_	0.3950	2	0.0432	11
			C_52_	0.6050	1	0.0662	1
C_6_	0.0925	8	C_61_	0.1833	3	0.0170	25
			C_62_	0.3376	2	0.0312	16
			C_63_	0.4790	1	0.0443	8
C_7_	0.0973	7	C_71_	0.1515	4	0.0147	28
			C_72_	0.1651	3	0.0161	26
			C_73_	0.3578	1	0.0348	13
			C_74_	0.3255	2	0.0317	14
C_8_	0.1069	6	C_81_	0.1256	5	0.0134	30
			C_82_	0.2056	4	0.0220	23
			C_83_	0.2095	3	0.0224	22
			C_84_	0.2310	1	0.0247	20
			C_85_	0.2283	2	0.0244	21

Note. The confidence level of weights is $\frac{1}{k}\overset{k}{\underset{i=1}{\sum }}\frac{\left|w_{i}^{31}-w_{i}^{31-1}\right|}{w_{i}^{31}}\times 100\%=0.7\%<5\%$ , i.e., significant confidence is 99.3%.

Overall, the “Relationship with patients (*C*_1_)” dimension was the most important, with an average weight level of 0.1731, followed by “Salary and benefit (*C*_4_)” and “Relationship with peer (*C*_2_)”. Relative to the other dimensions, “Relationship with organization (*C*_6_)” was the least important. “I am treated as a professional colleague by physicians (*C*_52_)” was the most important criterion, with an average global weight of 0.0662, while “At times I am overwhelmed by all the work I have to do (*C*_81_)” was the least important, with an average global weight of 0.0134.

### The Job Satisfaction Performance

**FIGURE 3:**
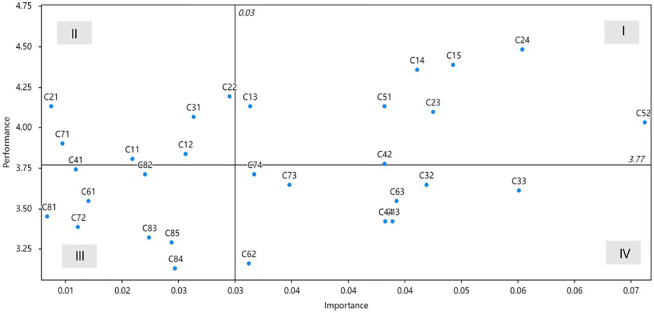
The quadrant diagram analysis for home healthcare nurses’ job satisfaction (China, 2022).

The IPA results of HHNs job satisfaction are summarized in Table 4 and illustrated in Figure 3.

**TABLE 4:** The important-performance analysis results for job satisfaction performance (China, 2022).

Criteria	Importance	Performance	Quadrant
Relationship with patients (*C*_1_)			
Patients are satisfied with the care that I provide (*C*_11_)	0.0209	3.8065	II
The relationships that I have established with patients are rewarding (*C*_12_)	0.0256	3.8387	II
I have helped patients maintain or improve their quality of life (*C*_13_)	0.0313	4.1290	I
My work is important and worthwhile (*C*_14_)	0.0461	4.3548	I
The patient care that I provide adheres to my professional standards (*C*_15_)	0.0493	4.3871	I
Relationship with peer (*C*_2_)			
The support I have from my nursing peers is a positive aspect of my job (*C*_21_)	0.0137	4.1290	II
I can communicate comfortably with the nurses I work with (*C*_22_)	0.0295	4.1935	II
There is a good amount of collegiality among the nurses I work with (*C*_23_)	0.0475	4.0968	I
I have peers whom I can rely on and turn to if necessary (*C*_24_)	0.0554	4.4839	I
Professional pride (*C*_3_)			
If I had to do it over again, I would choose home healthcare as my area of practice (*C*_31_)	0.0263	4.0645	II
I would commend my job to another health care professional (*C*_32_)	0.0469	3.6452	IV
I am proud to talk to people about the work I do (*C*_33_)	0.0551	3.6129	IV
Salary and benefit (*C*_4_)			
My present salary is satisfactory (*C*_41_)	0.0159	3.7419	III
An upgrading of the pay scales at this agency is needed (*C*_42_)	0.0432	3.7742	I
Nursing salaries at other agencies are better than salaries at this agency (*C*_43_)	0.0439	3.4194	IV
The benefit package at this agency is satisfactory to me (*C*_44_)	0.0433	3.4194	IV
Relationship with physician (*C*_5_)			
Physicians value my input on the status of their home healthcare patients (*C*_51_)	0.0432	4.1290	I
I am treated as a professional colleague by physicians (*C*_52_)	0.0662	4.0323	I
Relationship with organization (*C*_6_)			
I am satisfied with the professional relationship that I have with nursing administration at this agency (*C*_61_)	0.0170	3.5484	III
I have the power to generate change in organizational policy at this agency (*C*_62_)	0.0312	3.1613	IV
I have the opportunity to grow and develop as a professional nurse within this agency (*C*_63_)	0.0443	3.5484	IV
Autonomy and control (*C*_7_)			
I am able to adjust the hours of my work if needed (*C*_71_)	0.0147	3.9032	II
I have more flexibility in my hours of work than nurses in other practice settings (*C*_72_)	0.0161	3.3871	III
I have sufficient control over scheduling my time (*C*_73_)	0.0348	3.6452	IV
I have the independence to make important decisions in my day-to-day work (*C*_74_)	0.0317	3.7097	IV
Stress and workload (*C*_8_)			
At times I am overwhelmed by all the work I have to do (*C*_81_)	0.0134	3.4516	III
I could deliver better patient care if I had more time (*C*_82_)	0.0220	3.7097	III
I am able to meet the demands of my job (*C*_83_)	0.0224	3.3226	III
I am able to cope with the increased demands for documentation in home care (*C*_84_)	0.0247	3.1290	III
Sometimes I get frustrated because all my activities are programmed for me (*C*_85_)	0.0244	3.2903	III

Quadrant I includes “I have helped patients maintain or improve their quality of life (*C*_13_),” “My work is important and worthwhile (*C*_14_),” “The patient care that I provide adheres to my professional standards (*C*_15_),” “There is a good amount of collegiality among the nurses I work with (*C*_23_),” “I have peers whom I can rely on and turn to if necessary (*C*_24_),” “An upgrading of the pay scales at this agency is needed (*C*_42_),” “Physicians value my input on the status of their home healthcare patients (*C*_51_)”, and “I am treated as a professional colleague by physicians (*C*_52_).”

Quadrant II includes “Patients are satisfied with the care that I provide (*C*_11_),” “The relationships that I have established with patients are rewarding (*C*_12_),” “The support I have from my nursing peers is a positive aspect of my job (*C*_21_),” “I can communicate comfortably with the nurses I work with (*C*_22_),” “If I had to do it over again, I would choose home healthcare as my area of practice (*C*_31_),” and “I am able to adjust the hours of my work if needed (*C*_71_).”

Quadrant III includes “My present salary is satisfactory (*C*_41_),” “I am satisfied with the professional relationship that I have with nursing administration at this agency (*C*_61_),” “I have more flexibility in my hours of work than nurses in other practice settings (*C*_72_),” “At times I am overwhelmed by all the work I have to do (*C*_81_),” “I am able to meet the demands of my job (*C*_83_),” “I am able to cope with the increased demands for documentation in home care (*C*_84_),” and “Sometimes I get frustrated because all my activities are programmed for me (*C*_85_).”

Quadrant IV includes “I would commend my job to another health care professional (*C*_32_),” “I am proud to talk to people about the work I do (*C*_33_),” “Nursing salaries at other agencies are better than salaries at this agency (*C*_43_),” “The benefit package at this agency is satisfactory to me (*C*_44_),” “I have the power to generate change in organizational policy at this agency (*C*_62_),” “I have the opportunity to grow and develop as a professional nurse within this agency (*C*_63_),” “I have sufficient control over scheduling my time (*C*_73_),” and “I have the independence to make important decisions in my day-to-day work (*C*_74_).”

## Discussion

### Clinical Practice

HHNs provide skilled patient care and spend most of their time interacting with them. A relationship is established when nurses care for patients [43], and direct patient care can be rewarding and give a sense of value to nurses [44]. Nurses’ job satisfaction is positively correlated with the quality of care they provide. Improving the health status of patients and having a positive impact on patients are related to higher job satisfaction [45]. The degree to which a job reflects an individual’s values affects job satisfaction. Therefore, a sense of value and job identity plays an important role in nurses’ job satisfaction [46]. In addition, interactions with peers and physicians—teamwork, group cohesion, respect, and acknowledgment by physicians—are important job satisfaction-related factors [47, 48]. Remuneration is an important extrinsic factor affecting job quality and satisfaction [46], and many nurses recommend increasing remuneration as the most relevant financial incentive [49]. Therefore, these attributes (C_13_, C_14_, C_15_, C_23_, C_24_, C_42_, C_51_, and C_52_) have higher relative importance and priority than those in quadrant III. This indicates that home-healthcare-agency administrators should focus primarily on these attributes as they are the critical factors influencing HHNs job satisfaction.

Compared to other attributes, the attributes in quadrant IV (C_32_, C_33_, C_43_, C_44_, C_62_, C_63_, C_13_, C_73_, and C_74_) take precedence in improving job satisfaction performance in home healthcare nursing. Professional pride is key to nursing job satisfaction due to the positive image of nursing and pride in nursing skills. Threatening professional pride may lead to nurses choosing not to continue with nursing and not recommending the field to others [50]. Although remuneration was considered an important influencing factor for job satisfaction, the focus was on increasing salary and benefits to recruit and retain nurses. This may be a short-sighted approach as administrators must be mindful of pay equity [46, 51]. The opportunity for professional growth and influence on changes in organizational policy can help increase nurses’ job satisfaction [47, 52]. However, administrators limited by hospital budgets seldom provide sufficient time or opportunities for nurses seeking professional development [44]. Autonomy was defined as the ability to arrange one’s own schedule by prioritizing tasks, working alone, and having the freedom to make decisions within the nursing scope. It was considered a key factor affecting job satisfaction [46, 53]. Nurses in home healthcare settings have a higher level of autonomy than those in hospital-based settings [54]. However, because nurses in China do not exercise autonomous practices, the performance of the attributes *C*_73_ and *C*_74_ is poor [55].

The main findings of the study are as follows. First, we obtained the attribute weights of HHNJS and found that *C*_1_ is the most important factor influencing HHNs’ job satisfaction. Second, we evaluated the job satisfaction of HNNs and found that the HHNs were not satisfied with the attributes in quadrants III (*C*_41_, *C*_61_, *C*_72_, *C*_81_, *C*_83_, *C*_84_, *C*_85_) and IV (*C*_32_, *C*_33_, *C*_43_, *C*_44_, *C*_62_, *C*_63_, *C*_13_, *C*_73_, *C*_74_). Combined with the attribute weights, home-healthcare-agency administrators should focus their attention and resources on the attributes in quadrant IV.

### Limitations

This study has a few limitations. First, the study used the optimized CFPR method based on the AHP method to explore the relative weights of attributes and identify key factors. Although CFPRs can overcome the shortcomings of AHP and improve its practical value effectively, they are still based on the independent relationship between attributes. Second, this study was a cross-sectional survey, limited by its self-report nature, and might not reflect the long-term HHNs job satisfaction performance. Finally, the study only included 31 HHNs in one prefecture-level city in China; therefore, the findings cannot be widely generalized. Future research using random selection over a wider group of nurses from different regions is recommended.

### Conclusion

This study used the hybrid MCDM model to investigate the critical factors and performance of HHNs job satisfaction based on nurse experiences in three community hospital. The findings can help home-healthcare-agency administrators better understand the critical factors influencing the HHNs job satisfaction and the current job satisfaction performance. This could enable them to establish reasonable strategies to improve job satisfaction and retain the nursing workforce.
