# Supplementary material for: The Hybrid Multiple-Criteria Decision-Making Model for Home Healthcare Nurses’ Job Satisfaction Evaluation and Improvement
Source: Int J Public Health. 2022 Sep 28;67:1604940. doi: 10.3389/ijph.2022.1604940 (PMC9554012; doi:10.3389/ijph.2022.1604940)
Supplement: Supplementary file 1 [file DataSheet1.docx]

**The CFPRs Method**

The calculating procedure for the CFPRs is shown below:

Step 1: Construct an assessment system with *k* attributes. The HHNJS tool was used in our study. Therefore, there were eight attributes in the assessment system (*k* = 8).

Step 2: Apply 9-point scales to obtain expert opinions about the degree of preference relationship between the attributes. From this, we can establish a multiplicative preference set $R=\left(r_{ij}\right)$, where $r_{ij}\in\left[1/9,9\right]$ for each expert, as shown in Equation (1).

$r=\{r_{12},r_{23},...,r_{(k-1)k}\}$ (1)

Step 3: Use Equations (2)-(6) to construct the multiplicative preference set $R=\left(r_{ij}\right)$ into a consistent fuzzy preference relation matrix,$P=\left[x_{ij}\right]_{k\times k}$.

$s_{ij}=y(r_{ij})=\frac{1}{2}(1+\log_{9}r_{ij})$ (2)

$s_{ij}+s_{ji}=1\text{ }\forall i,j\in\left\{1,...,k\right\}$ (3)

$s_{ij}+s_{jn}+s_{ni}=\frac{3}{2}\text{ }\forall i<j<n$ (4)

(5)

$f(s_{ij})=\frac{s_{ij}+\varepsilon}{1+2\varepsilon}$ (6)

$\varepsilon$ should be converted into an absolute value and represents a minimal value in $s_{ij}$.

Step 4: Calculate the average weight of the fuzzy preference relation matrix$P=\left[x_{ij}\right]_{k\times k}$ using the normalized column method, as shown in Equation (7).

$W_{i}=\frac{1}{k}\sum_{j=1}^{k} \frac{x_{ij}}{\sum_{i=1}^{k} x_{ij}}$ (7)

Step 5: The results of the pairwise comparison matrix for each expert were identical. Therefore, we applied Equation (8) to calculate the degree of consensus in the expert group to ensure that the weight is robust and consistent.

$\frac{1}{k}\sum_{i=1}^{k} \frac{|w_{i}^{\rho}-w_{i}^{\rho-1}|}{w_{i}^{\rho}}\times100\%$ (8)

$\rho$ is the total number of experts and $w_{i}^{\rho}$ is the mean weight of the attribute $i$.
